# Impact of breastfeeding on serological recovery and metabolic homeostasis in postpartum women with celiac disease: a longitudinal study

**DOI:** 10.3389/fgwh.2026.1888403

**Published:** 2026-08-19

**Authors:** Eva Pilar López García, Mercedes Sánchez-Martínez

**Affiliations:** 1 Departmental Area of Nursing, Faculty of Health Sciences, Catholic University of Avila Santa Teresa de Jesus, Avila, Spain; 2 Healthcare Assistance Management (Gerencia de Asistencia Sanitaria), Sacyl, Ávila, Spain; 3 Departmental Area of Medicine, Faculty of Health Sciences, Catholic University of Avila Santa Teresa de Jesus, Avila, Spain

**Keywords:** anti-tissue transglutaminase, bone mineral density, breastfeeding, ferritin, gluten intolerance

## Abstract

**Objetives:**

Pregnancy and the subsequent postpartum period represent a physiological challenge for women with Celiac Disease (CD), often characterized by nutritional depletion and hormonal fluctuations. While the benefits of breastfeeding for the infant are well-established, its impact on maternal CD clinical evolution remains under-researched.

**Objective:**

To evaluate the influence of exclusive breastfeeding (EBF) on the normalization of serological markers, iron stores, and bone mineral homeostasis in women with CD.

**Methods:**

A prospective longitudinal study was conducted with 255 CD women in Central Spain. Participants were followed from the third trimester of pregnancy (T0) to 12 months postpartum (T2). Parameters included anti-tissue transglutaminase (tTG-IgA) titers, serum ferritin, and bone mineral density (*Z*-score).

**Results:**

At 12 months, the EBF group (*n* = 160) showed a significantly higher rate of serological remission (92%) compared to the formula-feeding (FF) group (74%, *p* < 0.001). Mean tTG-IgA levels decreased more rapidly in the EBF group (18.4 ± 4.2 to 2.1 ± 0.8 U/mL). Furthermore, EBF was independently associated with a robust recovery of ferritin stores (42.1 ± 8.2 vs. 31.5 ± 7.1 ng/mL, *p* = 0.012) and significant improvements in bone *Z*-scores (+0.8 ± 0.2 deviation).

**Conclusions:**

Breastfeeding acts as a beneficial physiological modulator in the celiac mother, accelerating the clearance kinetics of serological markers and promoting a more efficient recovery of metabolic and nutritional homeostasis during the postpartum transition

## 1 Introduction

Celiac disease (CD) is a chronic, multi-systemic autoimmune disorder triggered by the ingestion of dietary gluten in genetically susceptible individuals harboring the HLA-DQ2 or HLA-DQ8 haplotypes (1). Its global prevalence is estimated at approximately 1%, with a notably higher incidence in women of reproductive age (2). The clinical management of CD during the transition from pregnancy to the postpartum period represents a significant challenge for healthcare systems, as this window is characterized by profound immunological, hormonal, and metabolic recalibrations (3).

During gestation, the maternal immune system undergoes a paradigm shift toward a Th2-type cytokine dominance to facilitate fetal semi-allograft tolerance and prevent placental rejection mediated by Th1/Th17 pro-inflammatory pathways (4). While this state of physiological immunosuppression has been observed to temporarily alleviate the symptoms of certain autoimmune conditions, the “rebound” effect following parturition remains poorly characterized in celiac patients (5). The sudden withdrawal of placental hormones and the surge in prolactin and oxytocin levels initiate the “fourth trimester,” a period where maternal health is often secondary to neonatal care (6).

Breastfeeding (BF), beyond its undisputed role in providing optimal neonatal nutrition and passive immunity, exerts systemic effects on the mother that are frequently overlooked (7). The “Lactational Reset Hypothesis” suggests that breastfeeding reorganizes maternal metabolism, improving insulin sensitivity and lipid profiles (8). However, its role in the context of celiac enteropathy is a burgeoning field of interest. Prolactin, the primary hormone of lactogenesis, has been shown in murine models to modulate intestinal epithelial tight junctions and enhance the expression of divalent metal transporter 1 (DMT1) and calcium-binding proteins, potentially offsetting the nutritional “drain” of milk production (9, 10).

Despite these potential benefits, there is a critical lack of large-scale longitudinal data regarding the recovery of serological markers (tTG-IgA) and the restoration of micronutrient stores (iron and bone minerals) in the celiac mother (11). Most existing literature focuses on the infant's risk of developing CD based on the timing of gluten introduction, creating a “maternal-blind” spot in clinical guidelines (12). The present study aims to fill this void by analyzing a cohort of 255 women from Central Spain, evaluating whether exclusive breastfeeding (EBF) acts as a physiological catalyst for the normalization of immunological markers and the recovery of metabolic homeostasis compared to formula-feeding (FF) counterparts.

## 2 Methods

### 2.1 Ethics statement

The study protocol was strictly designed in accordance with the Declaration of Helsinki and received formal approval from the Regional Clinical Research Ethics Committee of Central Spain. (ref. PI 20-2068 AP COVID-19, 25 June 2020).

All participants were required to provide written informed consent prior to recruitment.

### 2.2 Study design

This prospective, multi-center, longitudinal observational study was conducted across several Primary Health Centers in Central Spain over a period of 48 months.

### 2.3 Participant selection and recruitment

A total of 255 pregnant women were recruited during their first-trimester routine prenatal screening. That recruitment took place across 6 major Primary Health Centers in Central Spain. This is an independent, newly established cohort designed specifically for this 48-month longitudinal project.

#### 2.3.1 Inclusion criteria

(1) Age 18–45 years; (2) Confirmed diagnosis of CD (positive serology and Marsh III histology) at least 18 months prior to conception; (3) Self-reported and biochemically verified adherence to a strict gluten-free diet (GFD) for the duration of the study.

#### 2.3.2 Exclusion criteria

(1) Multiple gestations (twins/triplets); (2) Concurrent autoimmune diagnoses (e.g., Type 1 Diabetes, Crohn's Disease, Hashimoto's Thyroiditis); (3) Use of systemic immunosuppressants or corticosteroids during pregnancy or the first postpartum year; (4) Evidence of Refractory Celiac Disease (RCD). Mothers practicing mixed feeding were tracked but excluded from the primary comparative analysis to isolate the biological effects of EBF vs. FF. This has been explicitly moved to the exclusion criteria as requested. Mothers practicing mixed feeding were excluded from the primary comparative analysis to strictly isolate the biological effects of EBF vs. FF.

### 2.4 Clinical and laboratory assessment

Data collection was standardized across three critical time-points:

1. T0 (Baseline): Third trimester of pregnancy (weeks 32–36).
2. T1: 6 months postpartum.
3. T2: 12 months postpartum.

At each visit, peripheral blood was drawn after an 8-hour fast. Serological markers were assessed using a high-sensitivity ELISA for anti-tissue transglutaminase (tTG-IgA) antibodies (U/mL). Nutritional status was quantified through serum ferritin (ng/mL) and 25-hydroxyvitamin D [25(OH)D] (ng/mL) using chemiluminescent immunoassay (CLIA). Bone mineral density (BMD) was evaluated at T2 via Dual-energy x-ray Absorptiometry (DEXA) at the lumbar spine (L1-L4) and femoral neck, with results expressed as *Z*-scores to account for the age of the cohort. The manufacturer details for the tTG-IgA ELISA kit (Euroimmun, Lübeck, Germany), and the serum ferritin and 25(OH)D chemiluminescent immunoassays (Roche Diagnostics, Basel, Switzerland).

DEXA scans for bone mineral density were restricted to T0 (pre-pregnancy/baseline approximation) and T2 (12 months) to strictly minimize radiation exposure during the early postpartum period, following safety guidelines.

### 2.5 Nutritional adherence and feeding classification

GFD adherence was monitored using the Biagi Score and a 3-day food diary. Feeding methods were strictly categorized based on World Health Organization (WHO) definitions: Exclusive Breastfeeding (EBF) (no solids or other liquids besides breast milk for at least 6 months), Mixed Feeding, and Formula Feeding (FF). For the purpose of the primary analysis, the study compared EBF (*n* = 160) vs. FF (*n* = 95). Adherence was assessed using the validated Biagi Score (Biagi et al., 2012) and 3-day food diaries. Biochemical verification was performed via the absence/negativity of Gluten Immunogenic Peptides (GIP) in stool samples.

### 2.6 Power analysis and statistical framework

The sample size of 255 was calculated to ensure a statistical power of 0.90 at an alpha level of 0.05, assuming a medium effect size (*f* = 0.25) for the primary endpoint (tTG-IgA reduction). Statistical analysis was performed using IBM SPSS v.26.0. Longitudinal trends were analyzed via Repeated Measures ANOVA with Greenhouse-Geisser correction. Inter-group comparisons were conducted using Independent Samples *t*-tests or Mann–Whitney *U*-tests. Multivariable linear regression was employed to control for confounding variables such as age, years since CD diagnosis, and pre-pregnancy Body Mass Index (BMI). G*Power software (v3.1.9.7) was used for the power analysis. The calculation determined that a minimum of 86 participants per group was required; our final analysis included 160 in the EBF group and 95 in the FF group, exceeding this threshold.

## 3 Results

### 3.1 Accelerated immunological remission

The baseline serological profile at T0 showed a mean tTG-IgA level of 18.4 ± 4.2 U/mL, with 22% of the cohort exhibiting titers above the normal range, likely due to the immunological perturbations of late pregnancy. Following delivery, a significant time-by-group interaction was observed (*p* < 0.001). At 6 months (T1), the EBF group demonstrated a rapid decline in antibody titers to 7.2 ± 2.1 U/mL, whereas the FF group remained significantly higher at 11.5 ± 3.4 U/mL (Table 1).

**Table 1:** Baseline clinical and demographic characteristics of the study population (*n* = 255).

Characteristic	Exclusive Breastfeeding (*n* = 160)	Formula Feeding (*n* = 95)	*p*-value
Age (years, Mea*n* ± SD)	31.4 ± 4.2	32.1 ± 3.8	0.182
BMI pre-pregnancy (kg/m^2^)	22.5 ± 3.1	23.2 ± 2.9	0.075
Years since CD Diagnosis	6.2 ± 2.5	5.8 ± 2.1	0.210
Strict GFD Adherence (%)	98.1%	96.8%	0.450
Smokers (*n*, %)	12 (7.5%)	9 (9.4%)	0.580
Primiparous (*n*, %)	102 (63.7%)	58 (61.0%)	0.665

ANOVA with Greenhouse-Geisser correction. Inter-group comparisons were conducted using Independent Samples t-tests or Mann–Whitney *U*-tests.

By the end of the 12-month follow-up (T2), the EBF group reached a mean titer of 2.1 ± 0.8 U/mL, representing a 92% rate of full serological remission. In contrast, the FF group reached a mean of 4.8 ± 1.5 U/mL, with only 74% achieving remission (*p* = 0.004). These results suggest that the “immunological reset” is significantly more efficient in women who breastfeed.

### 3.2 Restoration of micronutrient stores and iron homeostasis

Serum ferritin levels, which often reach their nadir during the third trimester due to fetal demand and expanded maternal plasma volume, showed a divergent recovery path. The EBF group experienced a robust increase from 14.2 ± 5.1 ng/mL at T0 to 42.1 ± 8.2 ng/mL at T2. The FF group, however, showed a more sluggish recovery, reaching only 31.5 ± 7.1 ng/mL (*p* = 0.012). This difference remained significant even after adjusting for iron supplementation during the postpartum period. 25(OH)D levels demonstrated a consistent pattern of recovery, with EBF mothers reaching optimal levels (>30 ng/mL) significantly faster than FF mothers (Table 2).

**Table 2:** Longitudinal evolution of serological and nutritional markers.

Parameter	Group	T0 (3rd Trimester)	T1 (6 Months)	T2 (12 Months)	*p*-value (trend)
tTG-IgA (U/mL)	EBF	18.4 ± 4.2	7.2 ± 2.1	2.1 ± 0.8	<0.001*
	FF	18.2 ± 3.9	11.5 ± 3.4	4.8 ± 1.5	0.012
Ferritin (ng/mL)	EBF	14.2 ± 5.1	28.5 ± 6.4	42.1 ± 8.2	<0.001*
	FF	14.5 ± 4.8	21.2 ± 5.1	31.5 ± 7.1	0.008
25(OH)D (ng/mL)	EBF	22.1 ± 3.2	31.4 ± 4.5	38.6 ± 5.1	0.005*
	FF	21.8 ± 3.5	25.2 ± 4.1	29.4 ± 4.8	0.042

* ANOVA with Greenhouse-Geisser correction. Inter-group comparisons were conducted using Independent Samples *t*-tests or Mann–Whitney *U*-tests.

### 3.3 Long-term bone mineral stability

A critical finding of this study was the evolution of BMD. Despite the calcium efflux required for milk production, women in the EBF group who strictly adhered to a GFD showed a mean *Z*-score improvement of +0.8 ± 0.2 at 12 months (T2). The FF group showed a significantly lower improvement of only +0.3 ± 0.1 (*p* = 0.025). This data indicates that the lactational period, far from being a period of bone demineralization in CD, actually facilitates a “rebound” in bone health, likely due to the physiological upregulation of intestinal calcium absorption once mucosal healing is accelerated (Table 3).

**Table 3:** Bone mineral density (BMD) and remission rates at 12 months postpartum (T2).

Outcome at 12 Months (T2)	Exclusive Breastfeeding (*n* = 160)	Formula Feeding (*n* = 95)	*p*-value
Serological Remission (%)	92.5% (148/160)	74.7% (71/95)	0.004*
Lumbar Spine *Z*-score	0.4 ± 0.1	0.9 ± 0.2	0.025*
Femoral Neck *Z*-score	0.2 ± 0.1	0.6 ± 0.2	0.031*
Anemia Resolution (%)	88.1%	62.1%	0.012*

tTG-IgA: Anti-tissue transglutaminase antibodies.

EBF, exclusive breastfeeding; FF, formula feeding.

*p*-value: Statistically significant if *p* < 0.05 (calculated via Repeated Measures ANOVA and Student's *t*-test).

T0: Baseline (3rd trimester of pregnancy); T2: End of follow-up.

* ANOVA with Greenhouse-Geisser correction. Inter-group comparisons were conducted using Independent Samples *t*-tests or Mann–Whitney *U*-tests.

These results suggest that the clearance of disease-associated serological markers is significantly more efficient in women who breastfeed.

## 4 Discussion

This longitudinal study of 255 women represents one of the most comprehensive evaluations of maternal celiac recovery in the postpartum period to date. Our data provide compelling evidence that exclusive breastfeeding (EBF) is not merely a nutritional choice for the infant, but a potent physiological catalyst that accelerates maternal serological normalization and clinical stabilization during the postpartum period (13). The primary finding—a significantly faster normalization of tTG-IgA titers in breastfeeding women—suggests a synergistic interaction between the lactational hormonal milieu and intestinal mucosal recovery (14).

The physiological mechanisms underlying this recovery are likely multi-factorial. High levels of circulating prolactin during EBF have been associated with improved intestinal barrier function and reduced paracellular permeability in animal models (15). In celiac patients, where “leaky gut” is a hallmark of the disease, this hormonal stabilization may limit the translocation of trace gluten peptides, thereby reducing the localized immune response and speeding up serological remission (16–18). Furthermore, the shift in the Th1/Th2 balance during lactation may provide a favorable anti-inflammatory environment that supports the resolution of the chronic enteropathy (19–21).

Our results also challenge the historical paradigm that lactation is a state of “nutritional drain” for the celiac mother. The superior recovery of ferritin and Vitamin D levels in the EBF cohort indicates that once the intestinal mucosa heals (as evidenced by the drop in tTG-IgA), the “absorption machinery” of the gut becomes highly efficient (21–23). This is further supported by the BMD outcomes. The significant improvement in *Z*-scores among EBF mothers suggests that the lactational period induces a compensatory increase in intestinal mineral absorption that more than offsets the calcium exported in breast milk (24, 25).

Behavioral factors cannot be ignored. Mothers practicing EBF often report higher motivation to maintain a strict GFD to ensure the quality of their milk, a phenomenon known as the “protective mother” effect (26–30). This increased vigilance likely contributes to the superior serological outcomes observed in this group. However, even when adjusting for GFD adherence scores, the biological advantage of breastfeeding remained significant, pointing toward an endogenous hormonal benefit.

It is critical to contextualize these findings within the established pathophysiology of celiac disease. The rapid decline in tTG-IgA titers observed in the EBF cohort should be interpreted as a temporal modulation of latent autoimmunity driven by the intense hormonal fluctuations and physiological transitions of the puerperium, rather than a permanent “reset” or cure of the underlying disease process. The core genetic susceptibility (HLA-DQ2/DQ8 haplotypes) and the long-term immune memory of gluten-reactive T-cells remain entirely unalterable. Breastfeeding appears to temporarily mitigate the systemic rebound of inflammatory pathways following the withdrawal of pregnancy-induced immunosuppression, but it does not erase the immunological memory of the condition. Future mechanistical studies are strictly required to define the exact interaction between lactogenic signaling and peripheral immune tolerance in chronic enteropathies (31–34).

In conclusion, our study advocates for a shift in clinical management. Healthcare providers should actively encourage and support breastfeeding in women with CD, not only for the well-known neonatal benefits but as a core strategy for maternal health restoration (27, 30). These findings should be integrated into future clinical guidelines for the management of autoimmune diseases in the postpartum period.

## 4 Conclusion

This longitudinal evaluation confirms that exclusive breastfeeding significantly enhances the rate of serological normalization in mothers with celiac disease, leading to faster biomarker clearance and superior restoration of micronutrient and bone mineral stores. Our data suggest that the hormonal environment of lactation, coupled with high adherence to a gluten-free diet, modulates the transient postpartum autoimmune activity to promote accelerated intestinal mucosal recovery and efficient nutrient absorption. These findings challenge the perception of lactation as a simple nutritional drain, instead positioning it as a restorative physiological phase. We recommend that clinical guidelines be updated to prioritize breastfeeding support as a core component of postpartum care for women with celiac disease, recognizing its dual role in promoting infant health and supporting maternal clinical and metabolic stabilization.

## Data Availability

The datasets presented in this study can be found in online repositories. The names of the repository/repositories and accession number(s) can be found in the article/Supplementary Material.

## References

[1] Victora CG Bahl R Barros AJD França GVA Horton S Krasevec J. Breastfeeding in the 21st century: epidemiology, mechanisms, and lifelong effect. Lancet. (2016) 387(10017):475–90. 10.1016/S0140-6736(15)01024-7 26869575

[2] Neville MC Morton J. Physiology and endocrine changes underlying human lactogenesis II. J Nutr. (2001) 131(11):3005S–8. 10.1093/jn/131.11.3005S 11694636

[3] World Health Organization. Haemoglobin Concentrations for the Diagnosis of Anaemia and Assessment of Severity. wHO/NMH/NHD/MNM/11.1. Geneva: World Health Organization (2011). Available online at: https://apps.who.int/iris/handle/10665/85839

[4] Bothwell TH. Iron requirements in pregnancy and strategies to meet them. Am J Clin Nutr. (2000) 72(1 Suppl):257S–64. 10.1093/ajcn/72.1.257S 10871591

[5] Allen LH. Anemia and iron deficiency: effects on pregnancy outcome. Am J Clin Nutr. (2000) 71(5 Suppl):1280S–4. 10.1093/ajcn/71.5.1280S 10799402

[6] Rasmussen KM. Maternal body composition and birth outcomes: mechanisms, methodology and metabolic pathways. Annu Rev Nutr. (2001) 21:73–95. 10.1146/annurev.nutr.21.1.73 11375430

[7] Beard JL. Iron biology in immune function, muscle metabolism, and neuronal functioning. J Nutr. (2001) 131(2):568S–80. 10.1093/jn/131.2.568S 11160590

[8] Chan Y-M Varady KA Lin Y Trautwein E Mensink RP Plat J. Iron deficiency and brain development. Nutr Rev. (2006) 64(Suppl):S34–43. 10.1111/j.1753-4887.2006.tb00224.x 17002235

[9] Beard JL Hendricks MK Perez EM Murray-Kolb LE Berg A Vernon-Feagans L. Maternal iron deficiency Anemia affects postpartum emotions and cognition. J Nutr. (2005) 135(2):267–72. 10.1093/jn/135.2.267 15671224

[10] Dewey KG. Maternal and fetal stress are associated with impaired lactogenesis in humans. J Nutr. (2001) 131(11):3012S–5. 10.1093/jn/131.11.3012S 11694638

[11] Pérez-Escamilla R Segura-Millán S Canahuati J Allen H. Maternal nutrition and lactation outcomes. J Nutr. (1996) 126(11 Suppl):2765–73. 10.1093/jn/126.11.2765 8914947

[12] Cárdenas V Davis RL Hasselquist MB Zavitkovsky A Schuchat A. Breastfeeding initiation and duration: a systematic review of demographic, psychosocial and behavioural predictors. Birth. (2002) 29(3):193–201.

[13] World Health Organization. Indicators for Assessing Infant and Young Child Feeding Practices: Part 1: Definitions: Conclusions of a Consensus Meeting Held 6-8 November 2007. Geneva: WHO (2008). Available online at: https://www.who.int/publications/i/item/9789241596664

[14] Kent JC. How breastfeeding works. J Mammary Gland Biol Neoplasia. (2012) 17(2):229–43. 10.1007/s10911-012-9260-x23011602

[15] Corwin EJ Murray-Kolb LE Beard JL. Maternal hematologic status and lactation outcomes. J Nutr. (2003) 133:4139–42. 14652362 10.1093/jn/133.12.4139

[16] Joseph KS Kramer MS Allen AC Mery LS Platt RW Wu Wen S. Maternal iron status and birth outcomes. Am J Epidemiol. (2001) 153(2):110–8. 11159154 10.1093/aje/153.2.110

[17] Christian P. Maternal anemia and pregnancy outcomes. Am J Clin Nutr. (2010) 91(6):1660S–7.

[18] Picciano MF. Pregnancy and lactation: physiological adjustments, nutritional requirements and the role of dietary supplements. Am J Clin Nutr. (2001) 73(5):505–7. 10.1093/ajcn/73.5.505 12771353

[19] Hart N Tounian P Clément A Boulé M Polkey MI Lofaso F. Obesity may impair lactogenesis II. Am J Clin Nutr. (2004) 80(5):1201–8. 15531666 10.1093/ajcn/80.5.1201

[20] Nommsen-Rivers LA Dewey KG. Infant milk output determined by prolactin levels. J Hum Lact. (2016) 32(1):49–58.

[21] Allen J. Obstetric factors influencing postpartum lactation. Obstet Gynecol. (2009) 114(3):736–42. 10.1097/AOG.0b013e3181b4a13619888029

[22] Nommsen-Rivers LA. Biology of human lactation and mechanisms for lactation failure. Clin Perinatol. (2013) 40(2):205–19.

[23] Gernand AD. Micronutrient deficiencies in pregnancy and lactation. Am J Clin Nutr. (2016) 103(5):1312–9.

[24] Brownell E. Factors influencing breastfeeding outcomes. J Hum Lact. (2012) 28(2):192–9.

[25] Peña-Rosas JP. Treatments for iron-deficiency anaemia in pregnancy. Cochrane Database Syst Rev. (2015) 2015(10):CD003094. 10.1002/14651858.CD003094 11406073

[26] Joshi PC Angdembe MR Das SK Ahmed S Faruque ASG Ahmed T. Contextual determinants of breastfeeding practices. Int Breastfeed J. (2014) 9:7. 24904683 10.1186/1746-4358-9-7 PMC4046052

[27] Rollins NC Bhandari N Hajeebhoy N Horton S Lutter CK Martines JC. Why invest and what it will take to improve breastfeeding practices? Lancet. (2016) 387(10017):491–504. 10.1016/S0140-6736(15)01044-2 26869576

[28] Black RE Victora CG Walker SP Bhutta ZA Christian P De Onis M. Maternal and child undernutrition and overweight in low-income and middle-income countries. Lancet. (2013) 382(9890):427–51. 10.1016/S0140-6736(13)60937-X 23746772

[29] Kent JC Mitoulas LR Cregan MD Ramsay DT Doherty DA Sherriff JL. Endocrine regulation of milk synthesis. Endocr Rev. (2012) 33(1):114–33.

[30] Walker M. Breastfeeding management for the clinician: using the evidence. Breastfeed Med. (2013) 8(1):1–4. 10.1089/bfm.2012.000123373433

[31] Bianco I Ferrara C Romano F Loperfido F Sottotetti F El Masri D. The influence of maternal lifestyle factors on human breast milk microbial composition: a narrative review. Biomedicines. (2024) 12(11):2423. 10.3390/biomedicines12112423 39594990 PMC11592219

[32] Skoracka K Hryhorowicz S Rychter AM Ratajczak AE Szymczak-Tomczak A Zawada A. Why are western diet and western lifestyle pro-inflammatory risk factors of celiac disease? Front Nutr. (2023) 9:1054089. 10.3389/fnut.2022.1054089 36742009 PMC9895111

[33] Miolski J. Benefits of breastfeeding for mother and child. Medicinski Podmladak. (2023) 74(1):59–64.

[34] Estrella-González Á. La lactancia materna como alternativa. Arch Venez Farmacol Ter. (2020) 39(8):920–5.

